# Supplementary material for: Curated and harmonised transcriptomics datasets of interstitial lung diseases
Source: Data Brief. 2025 Oct 14;63:112139. doi: 10.1016/j.dib.2025.112139 (PMC12581653; doi:10.1016/j.dib.2025.112139)
Supplement: Supplementary file 1 [file mmc1.zip › Supplementary_material/RNA-seq/fastQC reports/GSE180415/TRIMMED_SRR15194700_2_fastqc.html]

TRIMMED\_SRR15194700\_2.fastq.gz FastQC Report 

FastQC Report

Wed 25 Oct 2023  
TRIMMED\_SRR15194700\_2.fastq.gz

## Summary

- Basic Statistics
- Per base sequence quality
- Per sequence quality scores
- Per base sequence content
- Per sequence GC content
- Per base N content
- Sequence Length Distribution
- Sequence Duplication Levels
- Overrepresented sequences
- Adapter Content

## Basic Statistics

| Measure | Value |
| --- | --- |
| Filename | TRIMMED\_SRR15194700\_2.fastq.gz |
| File type | Conventional base calls |
| Encoding | Sanger / Illumina 1.9 |
| Total Sequences | 47046201 |
| Sequences flagged as poor quality | 0 |
| Sequence length | 60-80 |
| %GC | 55 |

## Per base sequence quality

## Per sequence quality scores

## Per base sequence content

## Per sequence GC content

## Per base N content

## Sequence Length Distribution

## Sequence Duplication Levels

## Overrepresented sequences

| Sequence | Count | Percentage | Possible Source |
| --- | --- | --- | --- |
| GGGGGGGGGGGGGGGGGGGGGGGGGGGGGGGGGGGGGGGGGGGGGGGGGG | 84310 | 0.1792068184208965 | No Hit |
| GGTGGCGCGTGCCTGTAGTCCCAGCTACTCGGGAGGCTGAGGCTGGAGGA | 76917 | 0.1634924783831111 | No Hit |
| GGTGGCGCGTGCCTGTAGTCCCAGCTACTCGGGAGGCTGAGGTGGGAGGA | 72296 | 0.1536702187706931 | No Hit |
| GATCAATCGCCCCCGGGGGTGCCTCCGGGCTCCTCGGGGTGCGCGGCTGG | 67443 | 0.1433548268860221 | No Hit |
| TTTCTATAGTGTGTTACTAGAGAAGTTTCTCTGAACGTGTAGAGCACCGA | 60284 | 0.1281378702607677 | No Hit |
| CCGGGCTCCTCGGGGTGCGCGGCTGGGGGTTCCCTCGCAGGGCCCGCCGG | 58227 | 0.12376557248480063 | No Hit |
| GTTCTGGGCTGTAGTGCGCTATGCCGATCGGGTGTCCGCACTAAGTTCGG | 54104 | 0.11500184680161528 | No Hit |
| GCGGTGGCGCGTGCCTGTAGTCCCAGCTACTCGGGAGGCTGAGGCTGGAG | 53658 | 0.11405384251961172 | No Hit |
| CGGTGGCGCGTGCCTGTAGTCCCAGCTACTCGGGAGGCTGAGGCTGGAGG | 51026 | 0.10845934191370733 | No Hit |
| GCGGTGGCGCGTGCCTGTAGTCCCAGCTACTCGGGAGGCTGAGGTGGGAG | 49822 | 0.10590015546632554 | No Hit |
| CGGTGGCGCGTGCCTGTAGTCCCAGCTACTCGGGAGGCTGAGGTGGGAGG | 47678 | 0.10134293308826359 | No Hit |

## Adapter Content

Produced by FastQC (version 0.11.7)
